# Glassy Carbon Open-Celled Foams as a Reinforcement in Polymer Matrix Composites Dedicated for Tribological Applications

**DOI:** 10.3390/ma16051805

**Published:** 2023-02-22

**Authors:** Jerzy Myalski, Marcin Godzierz, Karolina Olszowska, Urszula Szeluga, Sławomira Pusz, Stanisław Roskosz, Hanna Myalska-Głowacka, Andrzej Posmyk

**Affiliations:** 1 Faculty of Materials Engineering, Silesian University of Technology, Krasińskiego 8 Street, 40-019 Katowice, Poland; 2 Polish Academy of Sciences, Centre of Polymer and Carbon Materials, M. Curie-Skłodowskiej 34 Street, 41-819 Zabrze, Poland; 3 Faculty of Transport and Aviation Engineering, Silesian University of Technology, Krasińskiego 8 Street, 40-019 Katowice, Poland

**Keywords:** carbon foam, glassy carbon, open-celled foam, polymer matrix composite, sliding wear

## Abstract

This work presents the results of a tribological examination of polymer matrix composites reinforced with carbon foams with different porosity. The application of open-celled carbon foams allows an easy infiltration process by liquid epoxy resin. At the same time, carbon reinforcement remains its initial structure, which prevents its segregation in polymer matrix. Dry friction tests, conducted under 0.7, 2.1, 3.5 and 5.0 MPa loads, show that higher friction load results in higher mass loss, but it strongly lowers the coefficient of friction (COF). The change in coefficient of friction is related to the size of the pores of the carbon foam. Open-celled foams with pores size below 0.6 mm (40 and 60 ppi), used as a reinforcement in epoxy matrix, allow to obtain COF twice lower than composite reinforced with 20 ppi open-celled foam. This phenomenon occurs due to a change of friction mechanisms. In composites reinforced with open-celled foams, general wear mechanism is related to destruction of carbon components, which results in solid tribofilm formation. The application of novel reinforcement, in the form of open-celled foams with stable distance between carbon components, allows the decrease of COF and the improvement of stability, even under a very high friction load.

## 1. Introduction

The reinforcement of polymeric materials with carbon materials of various structures to improve their tribological properties is well described in the literature [1,2,3,4]. As polymer matrices of composites with these fillers, epoxy resins are proposed due to the fact that they are characterized by low shrinkage and good adhesion to various materials, including carbon materials, are proposed. By the selection of appropriate epoxy resin and curing agent, a highly cross-linked polymer network with high thermal stability and mechanical strength can be obtained, which is also very important from the point of view of increasing requirements for tribological parameters as well as for the reliability and durability of sliding nodes. Especially in maintenance-free nodes, operating under conditions of dry friction or with poor lubrication, it is required to make them more reliable at increasing values of external loads, when significant increase of the temperature of the system occurs [5]. Until now, instead of traditional metallic materials or alloys, thermoplastic polymers and their composites, in particular engineering polymers, such as polyamide, polyethylene, polyoxymethylene, and special polymers: polytetrafluoroethylene, polyimide, and polyether ether ketone are used in this role [6]. However, the advantages of epoxy matrix are much better thermal and dimensional stabilities. Epoxy resins reinforced with the appropriate fillers show even better performance parameters. The addition of carbon materials with good thermal conductivity and thermal expansion allows to compensate for the weaknesses of epoxy resins, including the relatively low thermal conductivity. Modification of polymers with various fillers to obtain two- or multi-component composites is an attractive procedure/formula to improve key parameters because it does not require large changes in the technology and processing of the base polymer. However, the greatest advantage of the application of carbon components, additionally to improving the thermal resistance and mechanical characteristics of the final epoxy-based composites, is the formation of solid tribofilm [7,8,9,10,11,12]. Commonly applied carbon fillers are in the form of large graphite particles, short carbon fibers, glassy carbon particles, carbon black as well as nanocomponents such as carbon nanotubes or graphene nanoplatelets. Pikhurov et al. [13] revealed a beneficial influence of carbon nanofillers, i.a., fullerenes on mechanical and tribological properties on PA6 matrix. However, an addition of glassy carbon allows for significant simplification of the composite production compared to introducing nanoscale components. However, the foams ensure even distribution of reinforcement in the composite.

The biggest advantage of the application of carbon components in epoxy matrix composites is solid tribofilm formation [14,15,16,17,18,19,20,21]. Similar results may also be obtained using compounds with low shear modulus such as mica, hexagonal zinc sulfide, MoS_2_, WS_2_ or hexagonal BN (boron nitride). However, only carbon components (glassy carbon, graphite or carbon black) and polymer fillers (PTFE, PU) have densities similar to epoxy matrix and do not cause a significant increase of the mass of the final product. Their application allows to reduce the coefficient of friction value and limits the wear of composite in comparison to neat epoxy matrix. Khun et al. [20] proved that the application of 2 wt.% of graphene sheets reduces six times the wear of the composite in comparison to neat epoxy, whereas the addition of 1 wt.% reduces the composite wear by a factor of three, independently of the sliding speed used. Those results are comparable with the application of 10 wt.% of porous glassy carbon micro-fillers presented in our previous research [21].

The second most important benefit of the application of carbon fillers is the change in the wear mechanism. This effect cannot be obtained using thermoplastic fillers, because their hardness is similar to, or lower than, that of epoxy. The application of carbon fillers in the form of particles, fibers or fabrics leads to the increase of the composite hardness as well as limits the wear of composite as a result of the distance between carbon components. Szeluga et al. [22] reported that the application of higher weight amount of carbon micro-filler results in a lower coefficient of friction of epoxy-based composites reinforced with porous glassy carbon particles. Moreover, the lower distance between carbon components led to a limitation of the plastic deformation of the matrix during friction, resulting in a lower wear of the composite [22].

The application of ceramic and carbon foams in metal matrix composites is well known from the literature [7,8,9,10,11,12,13]. The usage of this type of reinforcement gives a number of advantages in comparison to particulate or fibrous fillers, especially in the meaning of tribological properties [14,23,24,25]. It was shown that alumina foams with porosity in the range of 65–85% strongly improve the wear properties of composite compared to pure matrix [26] and composites reinforced with Al_2_O_3_ particles or short fibres [27]. Cree et al. [28] observed similar effects of wear reduction for metal matrix composites reinforced with SiC foams. It was also shown that the application of carbon foams with porosity approximately 60 ppi—pores per inch (3 vol.%) gives similar wear properties as particulate-reinforced composites with 7.5 vol% of glassy carbon, as described in [29]. However, open-celled foams are not commonly applied in polymer matrix composites, due to their higher density than epoxy matrix, as well as relatively high porosity (85–97%). Ligoda-Chmiel et al. [30,31] manufactured interpenetrating phase ceramic-polymer composites using epoxy resin and alumina foam. Such composites had relatively low density (below 1.9 g/cm^3^) and compressive strength in the range 40–129 MPa, depending on the porosity of the foam. It is worth mentioning that polymer composites reinforced with open-celled foams may be an interesting material to apply as elements for acoustic absorption [30,31,32].

The aim of the presented paper is to determine the correlation between open-celled carbon foams (C_of_) pores sizes, which is the result of the mean distance between carbon elements, and the friction coefficient of epoxy-based composites in a wide range of counter sample pressure. The applied carbon foams had an open porosity in the range of 95–97%, which results in a similar weight amount of carbon in the composites. All results were compared with those of composites reinforced with 10 wt.% of porous glassy carbon micro-fillers.

## 2. Materials and Methods

**Figure 1:**
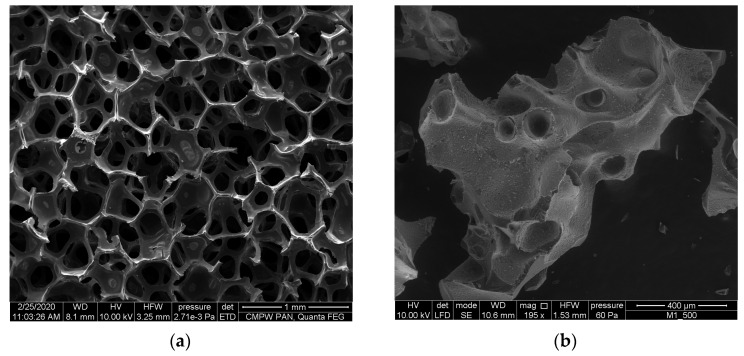
SEM micrographs of open-celled carbon foam with open porosity 60 ppi (**a**) and carbon foam with closed porosity (**b**).

As an epoxy matrix, the Epidian 6 epoxy resin (diglycidyl ether of bisphenol A, CIECH-Sarzyna, Poland) with amine curing agent (TETA, triethylenetetramine, CIECH-Sarzyna, Poland) was used. Open-porous glassy carbon foams (C_of_) obtained as a result of pyrolysis of polyurethane foams were the reinforcing components as open-celled foams with porosity 20, 40 and 60 ppi (Figure 1a). Subsequently, these polyurethane foams were coated with phenol-formaldehyde resin (FF110), which was subsequently cured under typical conditions for resin. Finally, the foams obtained were pyrolyzed under the standard conditions described elsewhere [33]. Epoxy composites with carbon open-celled foam reinforcement were fabricated using the infiltration method.

As a comparative material, the epoxy composites reinforced with closed-pore glassy carbon added to the matrix in the form of particles of up to 300 µm, obtained in the pyrolysis process of polymeric materials [21] marked as epoxy-CF, were used (Figure 1b). The method of manufacturing composites reinforced with glassy carbon micro-fillers is described elsewhere [33].

The microstructure of applied reinforcement was investigated using scanning electron microscope (SEM) Quanta FEI 250 FEG-SEM (Thermo Fischer Scientific, Waltham, MA, USA). The applied acceleration voltage was 10 kV and low vacuum mode was used. The different structures of the reinforcements applied are shown in Figure 1.

**Figure 2:**
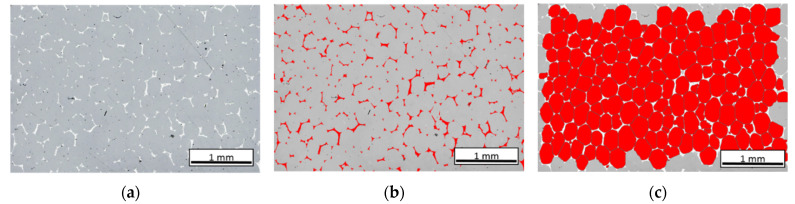
Automatic detection steps procedure of carbon foam cells and carbon skeleton in the LM image, (**a**) initial image, median filter, erosion 1 pp, (**b**) multiple binarization bright image (image (**a**)), removal of elements smaller than 100 pixels, (**c**) inversion image a, modified segmentation, manual correction remove frame, erosion with step 3.

The microstructure of polished composites was determined by light microscopy (Olympus GX-71) using the bright field technique. For quantitative stereological examination, 50 LM images were recorded giving a total area of 15 mm^2^. The ImageJ software was applied to the measurements, with the procedure presented in Figure 2.

**Figure 3:**
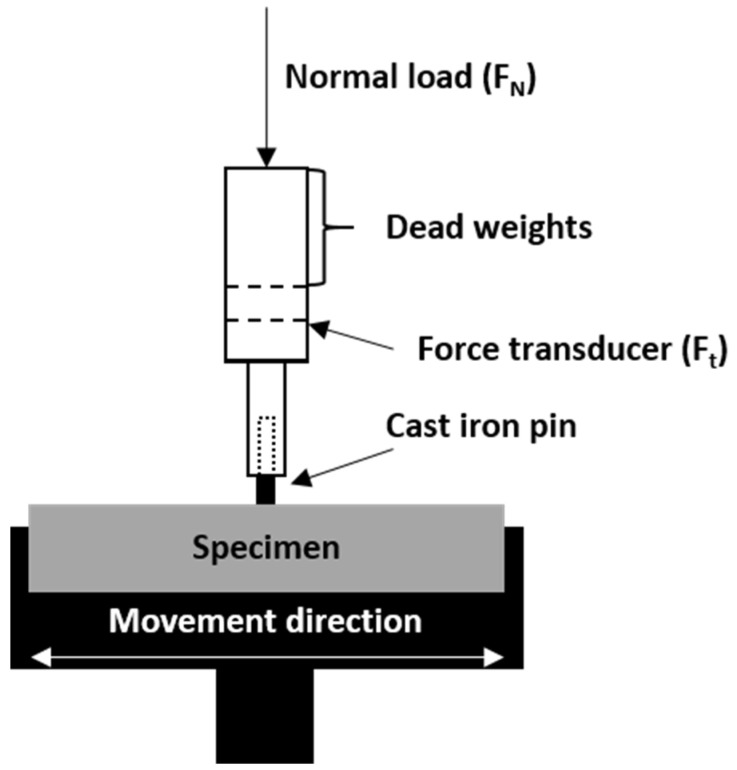
Schematic diagram of the pin-on-block TM-01M tribometer used in the tests.

The tribological characteristics were carried out under technically dry friction conditions using the pin-on-block method (reciprocating movement) using the TM-01M tribotester (Figure 3).

The velocity of 0.1 m/s was applied using a cast iron counter sample (ϕ = 3 mm) over a distance of 500 m with a pin load in the range of 5–35 N, which corresponds to the stress of 0.7–5.0 MPa. All tests were repeated at least three times to obtain reliable results. The surfaces of the samples after friction were examined using the FEI Quanta 250 FEG scanning electron microscope, in low-vacuum secondary electron technique, with an accelerating voltage of 5.0 kV.

## 3. Results and Discussion

### 3.1. Microstructure and Quantitative Analysis

**Figure 4:**
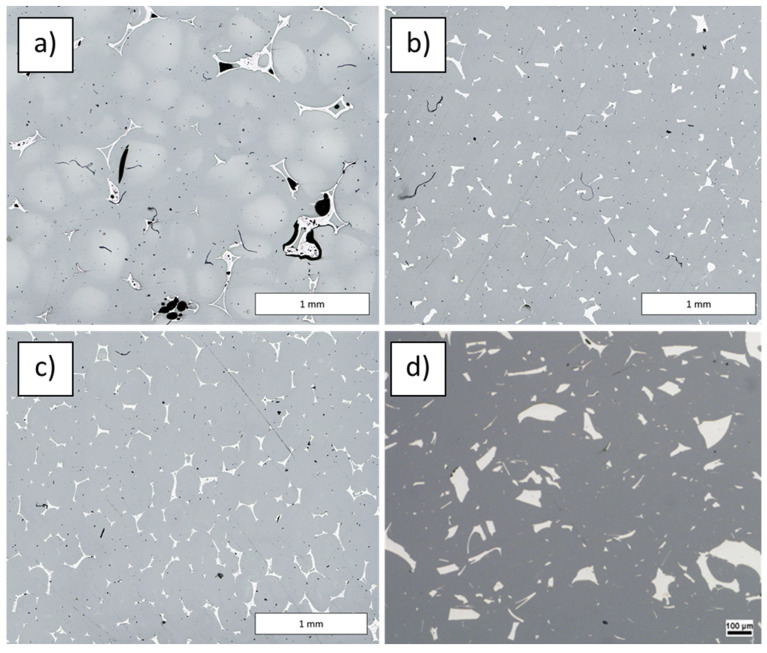
Microstructure of examined C_of_—epoxy composites with different foam porosity: (**a**) C_of_ 20 ppi, (**b**) C_of_ 40 ppi and (**c**) C_of_ 60 ppi, (**d**) CF with closed pores.

**Figure 5:**
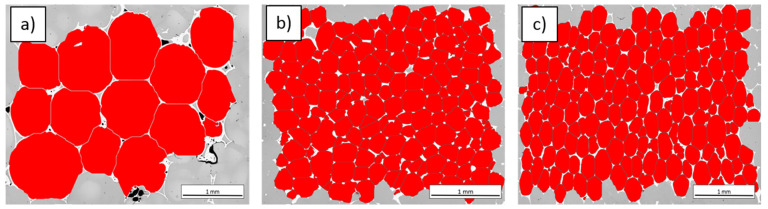
Detection of spheres in examined C_of_—epoxy composites with different foam porosity: (**a**) C_of_ 20 ppi, (**b**) C_of_ 40 ppi and (**c**) C_of_ 60 ppi.

The microstructure of examined carbon foam–epoxy composites (Figure 4) consists of carbon foam areas (white), micropores (black) and epoxy matrix (grey). Area fraction of pores in C_of_ 20 ppi—epoxy composite is higher than that of two other composites. Pores were detected also in carbon foam (CF) structure (Figure 4a). However they are partially filled in with the matrix. In composites with C_of_ 40 ppi and C_of_ 60 ppi, the pores in carbon foam structure are smaller, similar to those circular-shaped, gaseous ones in the matrix, and totally filled with epoxy (Figure 5).

Table 1 shows the stereological parameters calculated for examined composites. The highest mean area of plane section was observed for the composite with C_of_ 20 ppi, whereas the lowest one for the composite with C_of_ 60 ppi. Analysis of cells shape factors (δ and ξ, Table 1) indicates that the most spheroidal cells was observed for C_of_ 20 ppi, whereas for other composites the cells are slightly elongated (approx. 17%). Comparing mean values of distances between mass centers of cells, it should be noted that they are in good agreement with the nominal (theoretical) ppi values. The highest difference was observed for C_of_ 20 ppi (20%), slightly lower for C_of_ 40 ppi (15%), and the lowest for C_of_ 60 ppi (2%). Calculated ppi value based on image analysis is also in good agreement with nominal (theoretical) value.

The area fraction of the foam elements (Table 1) shows a slight increase (by approx. 0.5%), which is related to the number of elements on the analyzed cross-section and their average area of plane section. A significant increase in the number of foam elements on a cross-section of composite with C_of_ 40 ppi compared to that with C_of_ 20 ppi is the effect of a five-times-smaller average area of plane section.

Comparing the results of quantitative analysis, it should be noted that the difference between composites with C_of_ 40 ppi and C_of_ 60 ppi may not be significant for the possible tribological application, because the most important factor is an arrangement of reinforcement components [17], which is similar in above mentioned composites. The second important factor is quantity of reinforcement elements, which is approx. 17% lower for C_of_ 40 ppi than for C_of_ 60 ppi. Despite the difference in quantity, their areas of plane section are similar, but the area fraction is slightly higher for C_of_ 60 ppi).

### 3.2. Tribological Examinations

**Figure 6:**
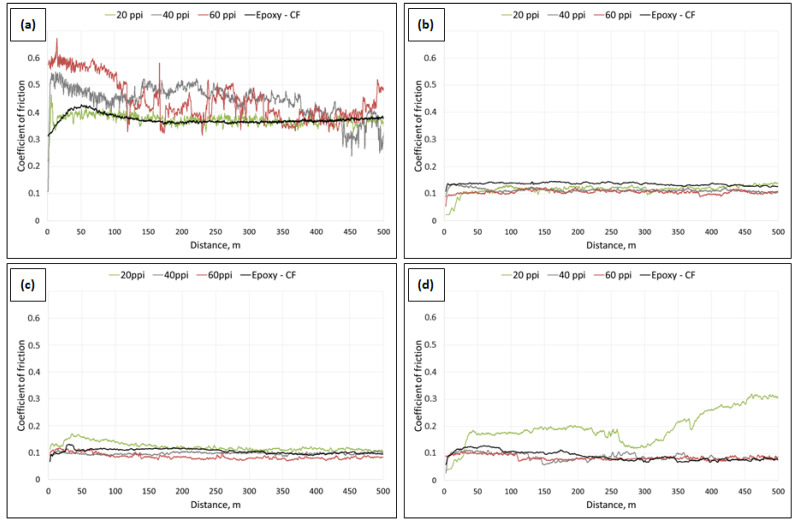
Coefficient of friction vs. sliding distance mean curves determined for neat epoxy and composites with carbon foams and carbon micro-fillers: (**a**) under 0.7 MPa load, (**b**) under 2.1 MPa load, (**c**) under 3.5 MPa load and (**d**) under 5.0 MPa load.

Coefficient of friction vs. sliding distance curves are presented in Figure 6, while mean values of COF and weight loss of samples are listed in Table 2. The lowest value of COF for 0.7 MPa pin load was obtained for epoxy—C_of_ 20 ppi and epoxy–CF. In the case of epoxy—C_of_ 40 ppi and C_of_ 60 ppi, the first stage of friction, in which the pair cooperation (lapping) takes place, was long and finished after the distance of 150 m. Most likely, this is the effect of the presence of hard carbon components and the distances between them. Moreover, the applied pressure of 0.7 MPa does not allow proper shear of glassy carbon particles, resulting in limited formation of solid tribofilm. Epoxy–CF and epoxy—C_of_ 20 ppi composites exhibit similar curve course of COF, however the wear of epoxy–CF is much lower. It is probably the result of a higher amount of carbon particles (two times higher than for epoxy—C_of_ 20 ppi) and four times lower distance between them.

Under the load of 2.1 MPa mean curves of COF vs. sliding distance of examined composites show similar trend, with shorter distance of lapping (around 50 m), followed by stable friction. The differences between the composites are negligible, about 0.02. However, despite similar mean COF values, the highest weight loss was observed for epoxy—C_of_ 20 ppi and the lowest for epoxy–CF.

Comparing coefficients of friction and weight loss of the composites examined under 3.5 MPa load, it should be mentioned that mean values of COF are on similar level, however for epoxy—C_of_ 20 ppi composite the longest distance of lapping was observed. Similarly, as under other loads, the lowest weight loss was denoted for epoxy–CF composite, six times lower than for epoxy—C_of_ 40 ppi and epoxy—C_of_ 60 ppi and eleven times lower than for epoxy—C_of_ 20 ppi composite.

Under the highest load, 5.0 MPa, composites reinforced with carbon foams 40 ppi and 60 ppi as well as with CF particles show similar curve course of COF vs. sliding distance. Only for epoxy—C_of_ 20 ppi composite the different curve course of COF vs. sliding distance was detected, which is the result of matrix plastificiation in reference to relatively high distance between carbon components on cross-section resulting in the presence of large areas of unreinforced epoxy matrix. During friction the temperature of cooperating pair increases, and those areas can cause adhesive contact with the pin, resulting in increasing coefficient of friction.

**Figure 7:**
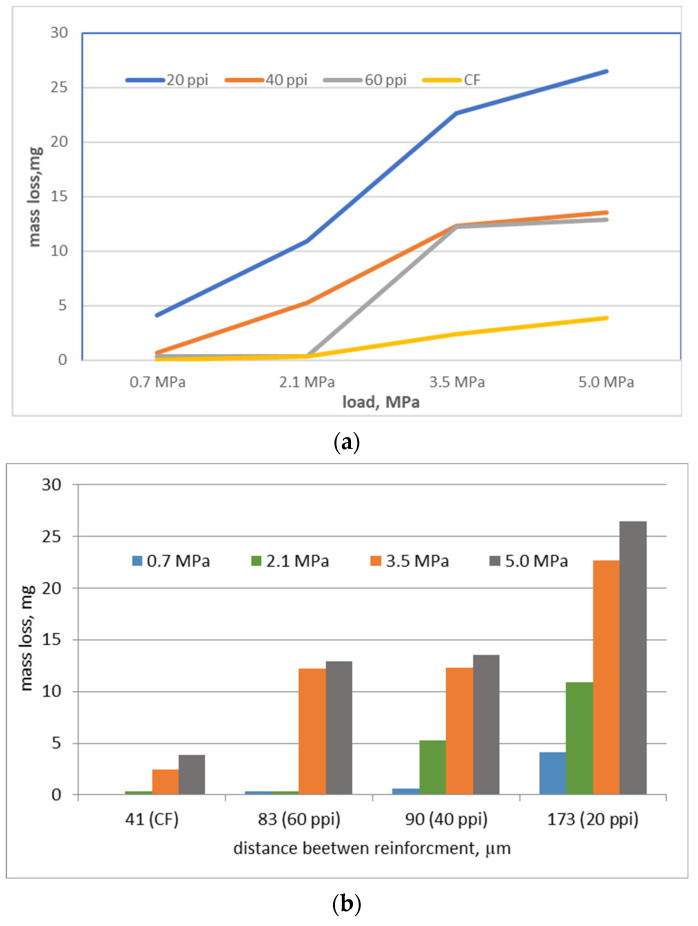
Correlation between mass loss vs. load (**a**) and mass loss vs. distance (**b**) between reinforcement carbon elements.

The wear results, defined as mass loss during friction, revealed that the wear of composite strongly depends on the size of the foam cells. For the composite containing the foam with the smallest porosity (20 ppi), the wear reaches the value several times higher than in the case of composites reinforced by the foams of 40 and 60 ppi. Although the differences in porosity between individual foams are the same, the mass loss of the composites with the foams of 40 and 60 ppi is similar (Figure 7a), which can be related to similar distances between the reinforcement elements in these composites (distances between the walls of the reinforcement cells). As can be seen in Figure 7b, small differences in the distances between the walls of the carbon reinforcement cells also cause a slight reduction in wear. Composites reinforced with CF foam, in which the distance between carbon particles is the smallest, are characterized by the lowest wear.

### 3.3. SEM Observation of the Surface after Friction Tests

**Figure 8:**
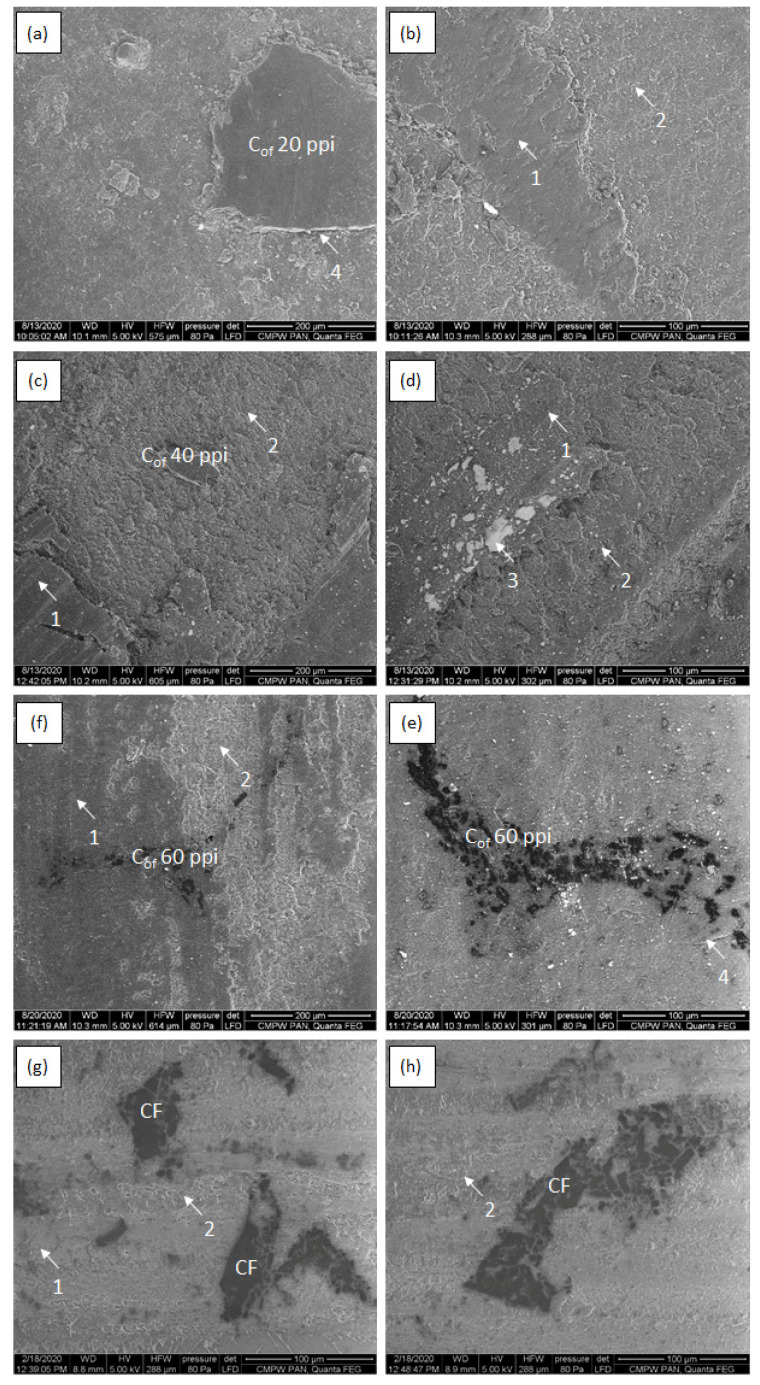
SEM micrographs of epoxy—C_of_ 20 ppi (**a**,**b**), epoxy—C_of_ 40 ppi (**c**,**d**),epoxy—C_of_ 20 ppi (**e**,**f**) and epoxy–CF (**g**,**h**) after cooperation with cast iron counter sample under 0.7 MPa load; 1—abrasive wear of carbon foam, 2—abrasive wear and adhesive shear and transfer of matrix, 3—solid tribofilm.

**Figure 9:**
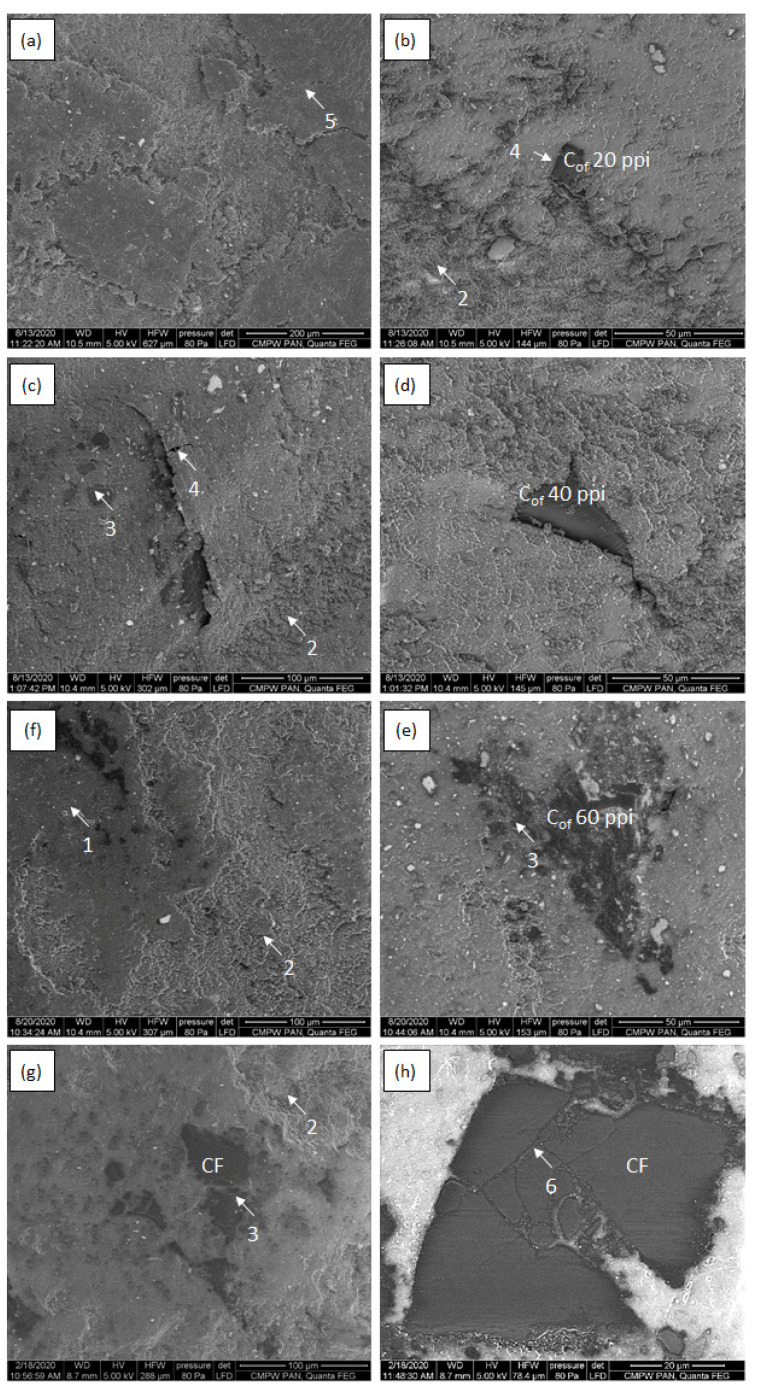
SEM micrographs of epoxy—C_of_ 20 ppi (**a**,**b**), epoxy—C_of_ 40 ppi (**c**,**d**),epoxy—C_of_ 20 ppi (**e**,**f**) and epoxy–CF (**g**,**h**) after cooperation with cast iron counter sample under 2.1 MPa load; 1—abrasive wear of carbon foam, 2—abrasive and adhesive wear and shear and transfer of matrix, 3—solid tribofilm, 4—cracks at interface, 5—matrix delamination, 6—cracks in glassy carbon structure.

**Figure 10:**
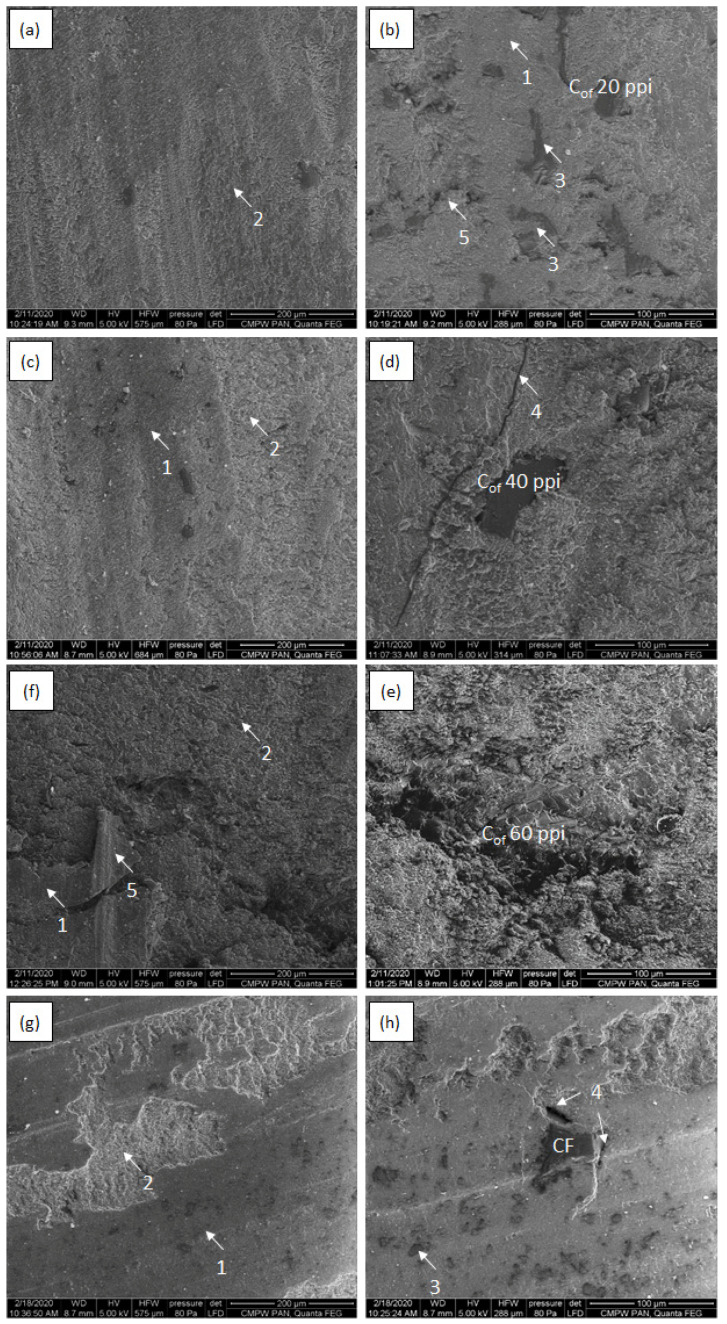
SEM micrographs of epoxy—C_of_ 20 ppi (**a**,**b**), epoxy—C_of_ 40 ppi (**c**,**d**),epoxy—C_of_ 20 ppi (**e**,**f**) and epoxy–CF (**g**,**h**) after cooperation with cast iron counter sample under 3.5 MPa load; 1—abrasive wear of carbon foam, 2—abrasive wear and adhesive shear and transfer of matrix, 3—solid tribofilm, 4—cracks at interface, 5—matrix delamination.

**Figure 11:**
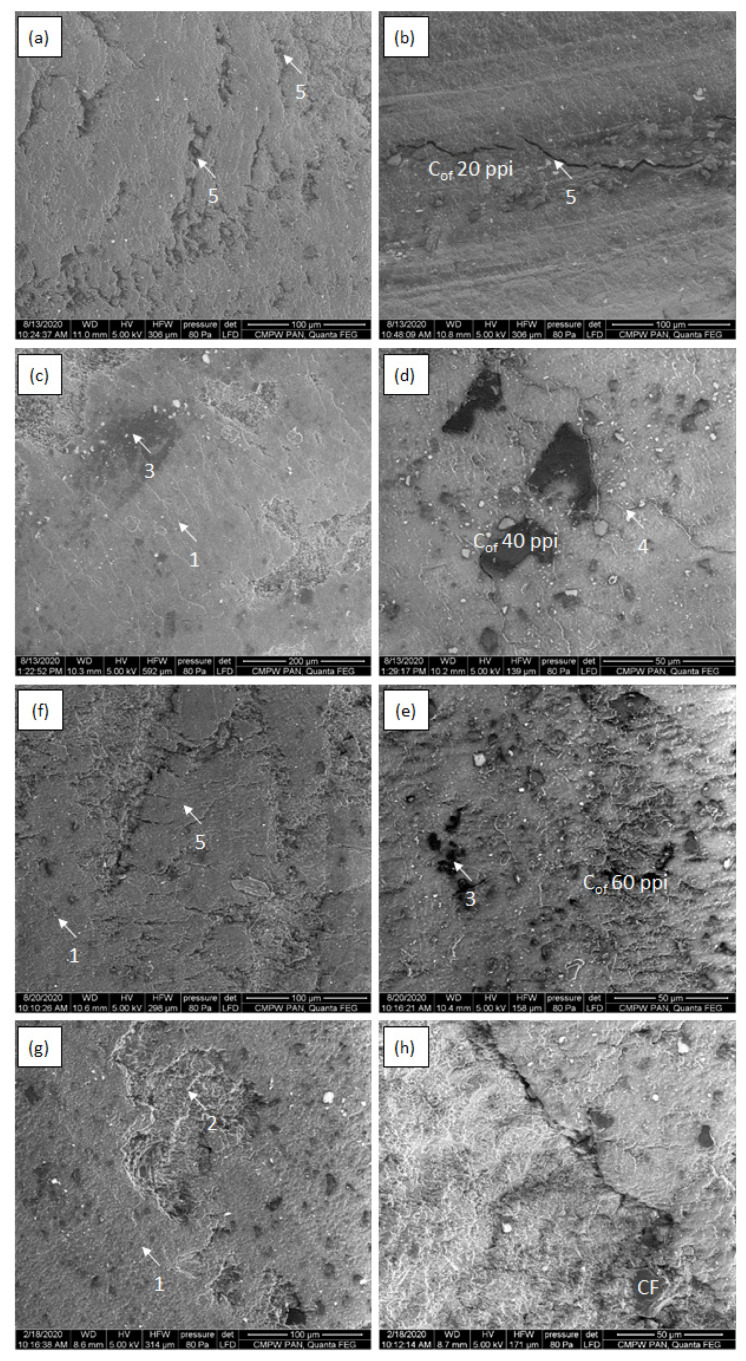
SEM micrographs of epoxy—C_of_ 20 ppi (**a**,**b**), epoxy—C_of_ 40 ppi (**c**,**d**),epoxy—C_of_ 20 ppi (**e**,**f**) and epoxy–CF (**g**,**h**) after cooperation with cast iron counter sample under 5.0 MPa load; 1—adhesive wear, 2—abrasive wear and adhesive shear and transfer of matrix, 3—solid tribofilm, 4—cracks at interface, 5—matrix delamination.

Figure 8, Figure 9, Figure 10 and Figure 11 shows SEM micrographs of the surfaces of the tested composites after cooperation with the cast iron counter-sample depending on the load in the friction node. The wear mechanisms change depending on the applied load and are dependent on the stereological parameters of the carbon components reinforcing the composites regarding the size of the foam pores diameters and the distances between the foam cell walls (Table 1). In the paper, the distance between the foam walls was characterized as the distance between the centers of gravity of the reinforcement elements (L_cf_). In the case of the low-porous 20 ppi foam, greater distance between the foam particles (about 170 µm) contributes mainly to the wear of the matrix material. With increasing diameters of the reinforcement cells, the matrix destruction area decreases, and occurs only in the areas between the carbon particles. Such a mechanism is particularly visible in the case of the friction surfaces in composites tested under low load (Figure 8 and Figure 9). Increasing load causes the appearance of much larger areas of foam degradation and wear (Figure 10 and Figure 11). As the load increases, the wear mechanism of the foam also changes. In the case of a small load (0.7 MPa), it can be seen that the epoxy matrix deforms plastically, and its plasticized fragments are torn off and removed from the friction surface, or in the form of deposition of wear products, they form large adhesive areas on the surface connected with the matrix (Figure 8a and Figure 9a). The load increase causes the adhesively formed layers of wear products to be removed from the friction surface (Figure 10f and Figure 11f). Areas of thermal degradation of the matrix material can be seen in places where the wear products have detached. With a greater load on the surface of epoxy matrix in the place of friction, the formation of wear products from the degradation of glassy carbon was observed. Carbon wear products bond adhesively to the friction surface limit the direct contact between the cast iron and the composite, creating a thin layer (carbon tribofilm) on the friction surface. This leads to a change in the type of materials cooperating in the friction node. In this case, the cast iron pin cooperates not with the epoxy-carbon layer but with the carbon layer, and the low value of the friction coefficient of the carbon material causes significant reduction of the friction coefficient (Figure 6) and the wear of the composite (Figure 7). The formation of a friction-friendly tribofilm (solid lubricant layer) on the surface of the composite is largely dependent on the structure of the reinforcing foam. In the case of low-porosity foams, the cell walls are much thicker than those of high porosity foams. The glassy carbon used in the composites is characterized by high hardness, which contributes to the reduction of wear. On the other hand, the low shear strength of carbon walls makes for the fragmentation of glassy carbon particles and the formation of thin layers of tribofilm. However, in order to obtain adequate glassy carbon fragmentation, an increase in the load in the friction process occurs, predisposing to the cracking and fragmentation of particles and the formation of carbon wear products. With low porosity of foam, greater distance between the particles and greater cell wall thickness of the foam, the load of 0.7 MPa is too small for glassy carbon degradation to occur. At a low load (0.7 MPa), only the appearance of small cracks and delamination of the matrix material was observed (Figure 8a,c). However, when the load is increased to 2.1 MPa (Figure 9), the surface after cooperation is smoother, which indicates the formation of a solid tribofilm. Locally, crumbled glassy carbon particles and cracks at the interface in the epoxy composite were detected. The load of 3.5 MPa and 5.0 MPa (Figure 10 and Figure 11) contributes to the increase in the degree of degradation and fragmentation of glassy carbon, it causes an increase in the number of cracks and the appearance of fine crushed carbon particles in the foam walls. The confirmation of the influence of the size of the load on the destruction of the glassy carbon structure are the results of the tests of the composite containing CF foam with closed porosity (Figure 8h). The large carbon particle only cracked under pressure and did not fragment into smaller grains.

Observations of wear mechanisms allow to write that they strongly depend on the porosity of the carbon foam used as reinforcement. Low porosity does not ensure the formation of favorable tribological properties, because the processes accompanying friction and wear are largely the result of matrix material wear processes. With greater porosity, the beneficial effect of the glassy carbon used as a reinforcing component is visible. The wear mechanism of carbon foams is comparable to wear mechanisms of graphite or MoS_2_. However, in the case of glassy carbon the structure of foam is amorphous, therefore the wear debris are much larger compared to commonly used solid lubricants with crystal structure.

## 4. Conclusions

The results presented in the paper allow us to determine the possibility the of application of novel carbon open-celled foams with 20, 40 and 60 ppi and open porosity in the range of 3–5% as a reinforcement in epoxy matrix composites dedicated for low frictional tribological applications. Obtained results suggest that the application of carbon open-celled foams led to obtaining a low coefficient of friction under higher loads than can be applied to a pure epoxy matrix. The porous structure of carbon open-celled foams results in decreasing amount of carbon reinforcement in the epoxy matrix, which allows obtaining of composites with similar tribological properties to one containing 10 wt.% of microporous carbon foams. Additionally, the application of carbon open-celled foams strongly improves the fabrication process, due to the lack of reinforcement agglomeration.

The tribological properties of C/epoxy composites can be designed considering the stereological characteristics of carbon open-celled foams and tribological test conditions. The coefficient of friction is reduced when C foam porosity increases. An increase in friction load results in significantly lower COF and its stability in friction time. Under 3.5 and 5.0 MPa loads coefficient of friction was about 0.1, while under 2.1 and 0.7 MPa loads were significantly higher, in the range of 0.2–0.4. Such changes in the COF of composites are related to different wear mechanisms. Under lower loads, the main wear mechanism is the abrasion of carbon and thermal-abrasive wear of the matrix. Higher loads result in the destruction of carbon reinforcement, resulting in solid tribofilm formation which decreases the coefficient of friction and wear of composites.

## Figures and Tables

**Table 1:** Calculated stereological parameters of examined composites.

	20 ppi	40 ppi	60 ppi	Epoxy–CF
Carbon component				
Area fraction; A_A_, %	3.76	4.25	4.89	7.31
Mean area of carbon plane section; A_cf_, μm^2^	3569	881	738	489
Distance between center of mass of carbon foam elements; L_cf_, μm	172	90	82	41
Quantity on 15 mm^2^ area	47	219	263	3128
Cells				
Dimensionless shape factor; δ	0.78	0.76	0.77	0.70
Dimensionless elongation factor; ξ	1.21	1.40	1.45	2.70
Calculated ppi value	15 ± 3	41 ± 4	50 ± 16	-
Quantity on 15 mm^2^ area	13	107	167	-

**Table 2:** Coefficient of friction and weight loss of sample determined for epoxy—C_of_ and epoxy—CF composites under different pin loads.

	20 ppi		40 ppi		60 ppi		Epoxy-CF	
Pressure	COF	Weight Loss of Sample, mg	COF	Weight Loss of Sample, mg	COF	Weight Loss of Sample, mg	COF	Weight Loss of Sample, mg
0.7 MPa	0.372 ± 0.024	4.15 ± 0.09	0.441 ± 0.059	0.64 ± 0.20	0.432 ± 0.080	0.34 ± 0.15	0.383 ± 0.025	0.064 ± 1.24 × 10^−6^
2.1 MPa	0.122 ± 0.021	10.89 ± 0.09	0.113 ± 0.014	5.24 ± 0.14	0.106 ± 0.013	2.68 ± 0.21	0.135 ± 0.007	0.33 ± 0.03
3.5 MPa	0.117 ± 0.018	22.66 ± 0.17	0.097 ± 0.010	12.32 ± 0.08	0.085 ± 0.013	12.20 ± 0.01	0.105 ± 0.011	2.43 ± 0.47
5.0 MPa	0.196 ± 0.063	26.48 ± 0.13	0.090 ± 0.019	13.54 ± 0.16	0.085 ± 0.013	12.90 ± 0.08	0.090 ± 0.018	3.86 ± 0.39

## Data Availability

Not applicable.
